# Changes in plant flammability‐related traits to fire regime characteristics and biomass conditions in the Cerrado

**DOI:** 10.1002/ajb2.70110

**Published:** 2025-10-14

**Authors:** Vagner Zanzarini, Davi R. Rossatto, Alessandra Fidelis

**Affiliations:** ^1^ Departamento de Ecologia Instituto de Biociências, Universidade de São Paulo (USP), São Paulo Brazil; ^2^ Lab of Vegetation Ecology, Instituto de Biociências Universidade Estadual Paulista (UNESP), Rio Claro Brazil; ^3^ Departamento de Biologia Faculdade de Ciências Agrárias e Veterinárias, Universidade Estadual Paulista (UNESP), Jaboticabal Brazil

**Keywords:** combustibility, consumability, fire frequency, fire history, fire spread, open savanna, plant dead biomass

## Abstract

**Premise:**

Flammability‐related traits in open savanna plant communities may shift in response to fire frequency (high vs. low) and history (recently vs. fire exclusion). Dead biomass accumulation and moisture content are expected to drive flammability components (combustibility and consumability). We hypothesized that in low fire frequency and fire exclusion areas, dead biomass accumulates, prolonging combustion duration with higher maximum temperatures and biomass consumption. Conversely, greater biomass should enhance combustibility, while higher moisture should dampen it.

**Methods:**

In Cerrado open savannas, we selected areas with high or low fire frequency and areas that were recently burned or excluded from fire for the last 21 years. For grasses, forbs, and shrubs, we measured the following flammability‐related traits: moisture content, dead biomass, burn rate, maximum fire temperature, and burned biomass.

**Results:**

Dead biomass remained similar between fire frequencies and histories. Plants burned slowly (~0.5 cm s^–1^) in areas where fire frequency was low or excluded. In all areas, ca 60% of the plant biomass was consumed by fire. The percentage of initial dead biomass increased the flammability components until 75% dead biomass, but beyond this threshold, burn rate, temperature, and burned biomass declined. Moisture content consistently reduced temperature and amount of biomass burned.

**Conclusions:**

Areas with fire excluded had slower fire spread but the amount of plant biomass consumed was not substantially lower. The amount of dead biomass has nonlinear relationships with combustibility and consumability, indicating that areas with more biomass accumulation may have lower flammability. Thus, we need to investigate how flammability‐related traits vary in plant communities under different fire regimes to understand fire behavior and improve management decisions.

## Graphical abstract

(A) Plant flammability‐related traits according to fire frequency and fire history in open savannas of the Cerrado. (B) The relationships between biomass conditions with flammability components.

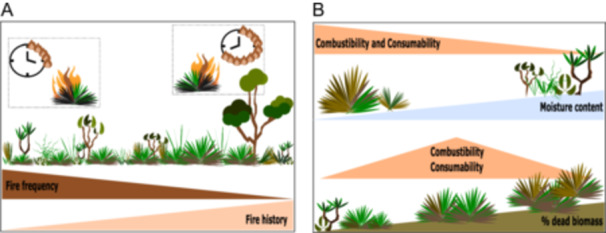

Fire‐prone ecosystems worldwide have distinct fire regimes, varying in intensity, frequency, season, and extent (Archibald et al., 2013). In tropical savannas, fire regimes are diverse (Simpson et al., 2022), with fires often being intense and spreading over large areas (e.g., Australian savannas; Archibald et al., 2013). In contrast, in savannas of Africa and South America (e.g., Cerrado), fires are not always intense and may affect small to large areas (Archibald et al., 2013). These differences in fire intensities are closely linked to specific grass traits dominating these systems (e.g., flammability traits, Simpson et al., 2022). Fire regimes play a crucial role in shaping functional syndromes of fire‐related traits, influencing the functional composition and diversification of plant communities. In turn, these changes in the vegetation can affect fire spread across the landscape (Bond and Van Wilgen, 1996; Keeley et al., 2011; Pausas and Bond, 2022; Simpson et al., 2022).

The way fire spreads and burns in an ecosystem can be assessed through the measurement of flammability, which has different components such as ignitability, combustibility, sustainability, and consumability (Anderson, 1970; Gill and Zylstra, 2005; Pausas et al., 2017). Plant flammability is characterized by a suite of traits that determine the burn capacity of plant organs or tissues in specific environmental conditions (Pausas et al., 2017). Therefore, studies on the relationship between fire regime characteristics and flammable components of the vegetation are essential for predicting fire behavior, assessing fire risk, and improving future decisions on fire management strategies.

In tropical savannas, grasses have the most‐flammable tissues among the diverse plant growth forms (e.g., trees, shrubs, forbs, and grasses) (Simpson et al., 2016; Zanzarini et al., 2022). In the Cerrado, fire typically consumes around 90% of the herbaceous aboveground biomass, particularly in open savannas (Rissi et al., 2017) because grasses are the major component of the fuel load (Coutinho, 1982). This high combustion efficiency is associated with specific traits in grass species that allow them to burn easily and quickly (Simpson et al., 2016; Archibald et al., 2019; Hempson et al., 2019; Zanzarini et al., 2022). Grasses often have aerated biomass (Archibald et al., 2019), which accumulates over the seasons. During the dry season, they rapidly convert live biomass into dead biomass (Bond, 2008; Linder et al., 2018), maintaining low water content in their tussocks compared to other growth forms (Zanzarini et al., 2022). These biomass conditions enhance the flammability of grasses compared to forbs and shrubs (Zanzarini et al., 2022). However, further investigations are needed to understand how the conditions in the herbaceous layer biomass (e.g., dead biomass accumulation due to the absence of fire) influence flammability, potentially increasing or decreasing the system's overall flammability.

When fire is absent from a savanna, some grass species accumulate large amounts of dead biomass (Cianciaruso et al., 2010; Rodrigues and Fidelis, 2022), creating dense fuels that affect fire spread and consumability (Simpson et al., 2016). Additionally, the reduced flammability of non‐graminoid species (Zanzarini et al., 2022), influences the fire behavior in the herbaceous layer. These species typically burn only when a highly flammable grassy component is present (Zanzarini et al., 2022). Thus, regular fire occurrences likely maintain the overall system's flammability by providing biomass conditions of the grassy layer that are suitable for fire spread and consumability.

Over the years, fire regimes have significantly changed across tropical savannas (Kelley et al., 2019; Bowman et al., 2020). Human activities and management decisions such as land‐use changes and zero‐fire policies (Durigan and Ratter, 2016; Bowman et al., 2020) have either increased (Lasslop and Kloster, 2017) or decreased the number of fire events (Andela et al., 2017). In the Cerrado, the natural fire interval has ranged from 1 to 5 years in the last decades (Eiten, 1972; Pivello, 2011), but changes in this interval are altering the fuel load and subsequent fire events (Pivello, 2011; Fidelis et al., 2018; Zupo et al., 2022). Changes in fire frequency can profoundly affect savanna vegetation structure and composition, particularly by modifying biomass accumulation patterns (Simpson et al., 2022; Zupo et al., 2022). For example, across savanna–forest transitions, flammability decreases as the grass layer is suppressed by increasing tree cover (Newberry et al., 2020). The shade environment reduces grass cover (Pilon et al., 2020), creating a more humid microclimate (Hoffmann et al., 2012b) and patches of exposed soil, which may disrupt the continuous fire propagation typical of open savannas (Archibald et al., 2013). In addition, the dominance of lower‐flammability grasses near the forest edge also influences fire behavior in transition areas (Cardoso et al., 2018).

While savannas excluded from fire may transition to closed ecosystems under favorable climatic and edaphic conditions (Abreu et al., 2017; Rosan et al., 2019), other areas may remain as an open savanna due to soil limitations on tree growth (Hoffmann et al., 2012a; Veldman et al., 2015). In these cases, fire exclusion leads to increased biomass accumulation in the grass layer (Fidelis et al., 2013; Zupo et al., 2022), which could elevate fire risk and alter fire intensity and spread (Zupo et al., 2022). Thus, understanding how biomass accumulation changes with fire regime characteristics and how these patterns influence flammability is crucial for predicting fire dynamics and developing effective fire management strategies.

Here we investigated changes in flammability‐related traits of herbaceous layer plants in response to fire frequency and fire history, determined by assessing the year since the last fire and the relationship between biomass conditions and flammability components in open savanna areas of the Cerrado. We addressed the following questions and hypotheses.

(1) Do plant flammability‐related traits (dead biomass, moisture content, burn rate, maximum temperature, and burned biomass) change according to fire frequency (high vs. low) and fire history (recently burned vs. fire excluded)?
  We hypothesized that in areas with low fire frequency or excluded from fire, dead biomass will accumulate, particularly due to the dominance of grasses in open savannas (Coutinho, 1982). In contrast, we expect areas with frequent fires and recent burns to have plants with higher moisture content due to the prevalence of live plant tissues. We also hypothesize that fire spread, maximum fire temperature reached during the burn, and biomass consumption will increase in areas with low fire frequency or exclusion, driven by the accumulation of dead biomass. These changes may enhance the overall flammability of open savannas.
(2) What is the relationship between plant biomass conditions (dead biomass and moisture content) and flammability components (burn rate, maximum temperature, and burned biomass), which are related to combustibility and consumability?
  We hypothesized that higher amounts of dead biomass will have higher combustibility and consumability because dead biomass is essential for fire propagation. Conversely, increased moisture content is expected to have lower combustibility and consumability because moist tissues are less flammable (Zanzarini et al., 2022).

## MATERIALS AND METHODS

### Study site

The study was conducted at the Reserva Natural Serra do Tombador (RNST, 13°35′38″ S, 47°45′51″ W, 8900 ha, 118 m a.s.l), Central Brazil, in open savannas of the Cerrado. The climate in the region is tropical, with a marked dry season between May to October. The mean annual rainfall is 1300–1500 mm, and the mean temperature is 20°–25°C (Antonelli‐Filho, 2011). These savannas are composed of a species‐rich herbaceous layer, dominated by C_4_ grasses, forbs, small shrubs, and sparse trees (Coutinho, 1982).

**Figure 1:**
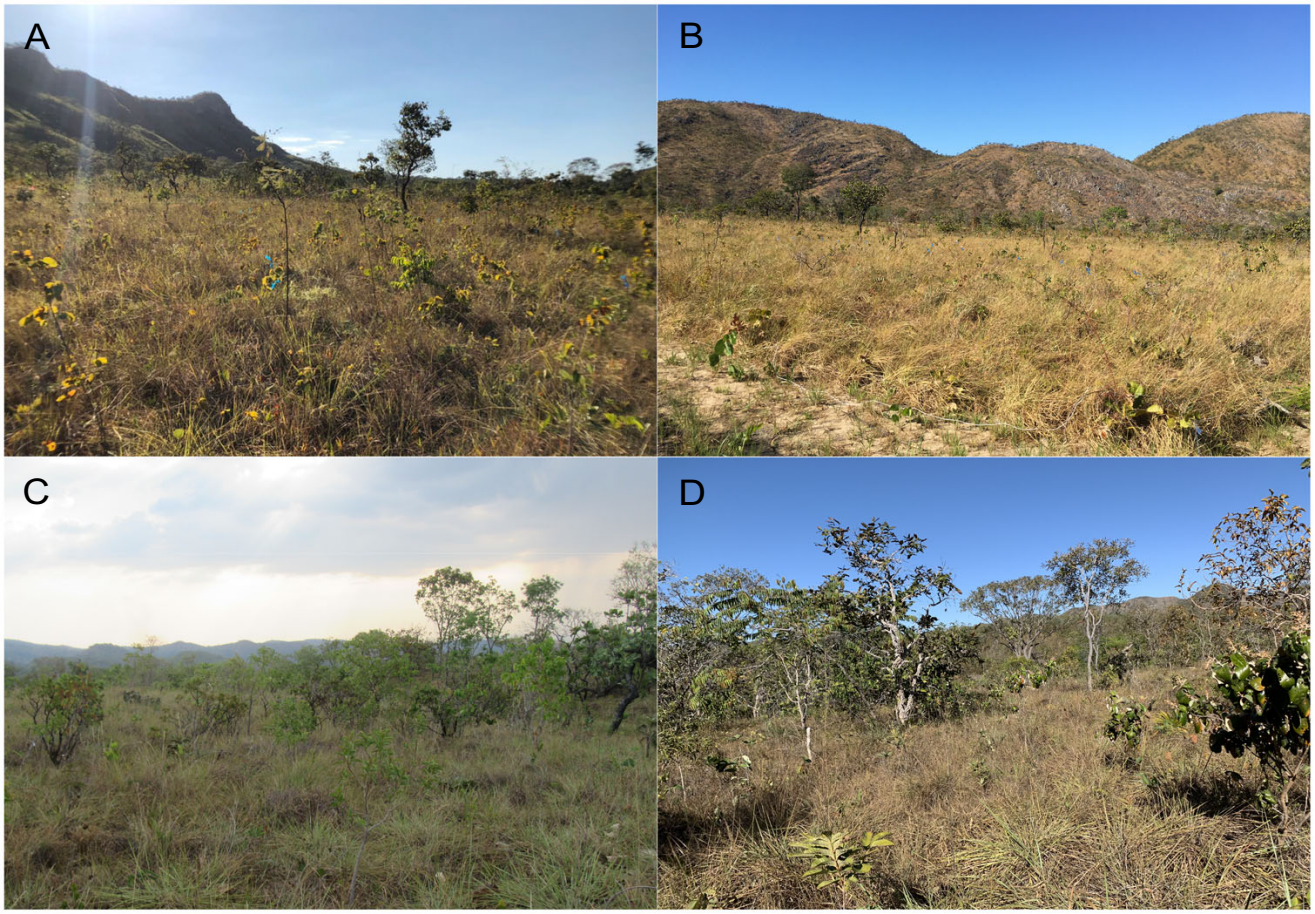
The four selected areas in open savannas of Cerrado in Central Brazil where plants were sampled to assess the plant flammability‐related traits of herbaceous layer species that represent 80% of total community abundance in each area. (A) High fire frequency, recently burned (burned in 2019); (B) high fire frequency, fire excluded since 2011; (C) low fire frequency, recently burned (burned in 2017); (D) low fire frequency, fire excluded since 2001.

To investigate whether plant flammability‐related traits change in relation to fire frequency (high or low) and fire history (recently burned or fire excluded), we selected four areas of open savannas at the RNST and sampled species traits at the end of September 2021. Then we determined the fire frequency (number of fire occurrences) and fire history (year since the last fire) of each area for the previous 21 years based on data previously extracted by our research team using Landsat imagery and manual vectorization of burn scars. We selected two areas that are part of a long‐term fire experiment established at the RNST in 2013, with experimental plots (30 × 30 m) subjected to different fire treatments (season and frequency; Rissi et al., 2017; Rodrigues et al., 2021). Fire events in the experimental area were natural until the establishment of the experiment (2011). After that, biennial prescribed burns were done at the end of the dry season. These two areas (ca 0.36 ha and 0.27 ha) were considered to have a high fire frequency, one with 11 fire events, the other with seven (Figure 1A, B). In relation to fire history, the last fire event in these two areas was in 2019 and 2011, respectively (Figure 1A, B). In general, Cerrado open savannas experience natural fires within 2.5 years, which can vary according to the region (Pivello, 2011); thus, we classified the selected areas as high fire frequency. The other two areas (each ca 0.36 ha) are not part of the long‐term fire experiment but are 3 km from the experimental area. We considered these areas as having low fire frequency; one had only two and the other just one natural fire in the last 21 years, with the last fire in 2017 and 2001, respectively (Figure 1C, D). In relation to fire history, we grouped areas where the last fire occurrence was in 2019 (Figure 1A) and 2017 (Figure 1B) as recently burned and areas with last fire occurrence in 2011 (Figure 1C) and 2001 (Figure 1D) as fire excluded. The exact year of each fire is provided in Appendix S1.

### Methods

#### Community composition and species selection

Within each selected area, we randomly established 30 1 × 1 m plots separated by at least 5 m, avoiding trees and anthills, and visually estimated the plant cover (%, as described by Wikum and Shanholtzer, 1978) for each species from the herbaceous layer (graminoids, forbs, and shrubs ≤1.5 m; Appendix S2), during the dry season (September 2021). We then selected the species that represented 80% of the entire community abundance in each area (Grime, 1998; Magurran, 2004) and measured flammability‐related traits. We measured the selected traits for 10 individuals/species, randomly chosen and sampled at least 3 m apart. We used five of these individuals for each species for plant traits that were measured in the laboratory (e.g., moisture content and dead biomass), and the other five individuals were burned in the field using equipment to measure flammability components (described below). During the burn experiments, we measured the remaining fire‐related traits (e.g., maximum fire temperature reached by plant material, burn rate, and burned biomass).

#### Trait measurements

We selected five flammability‐related traits and measured them at the individual level to assess the differences in the flammability of plants among the four areas. We measured the total individual moisture content (%) and the percentage of attached dead biomass (%) for five individuals per species. To calculate moisture content, we obtained the fresh mass of the aboveground biomass of each individual in the field and the dry mass in the laboratory, drying the samples at 80°C for 48 h. To obtain the percentage of dead biomass, we sorted the aboveground biomass into live and dead biomass for each individual in the lab and used the dry mass to calculate the percentage of dead material in relation to the total mass of each individual.

The other five individuals were burned separately in flammability tests to determine the maximum fire temperature reached (°C), burned biomass (%), and the burn rate (cm s^−1^) as described by Jaureguiberry et al. (2011), using a device that we constructed to measure flammability components in plant shoots (Jaureguiberry et al., 2011; Appendix S3). We measured the maximum fire temperature (°C) of plant samples using an infrared thermometer (Bosch GIS 1000 C, Robert Bosch Tool, Mount Prospect, IL, USA) directing it to the plant biomass during the burn process. For burned biomass (%), we visually estimated the individual biomass percentage consumed by fire after the burning process. We then obtained the burn rate (cm s^–1^) after measuring the length of the burnt portion of the shoot (cm) and the duration (s) of the burning process, dividing the length by duration for each plant (Jaureguiberry et al., 2011).

### Data analyses

To investigate whether plant flammability‐related traits varied in response to fire frequency (high vs. low) and fire history (recently burned vs. fire excluded), and their interaction, we constructed generalized linear mixed models. We used the function glmmTMB in the R package glmmTMB (Brooks et al., 2017) and constructed models for each response variable (moisture content, dead biomass, burn rate, maximum temperature, and burned biomass), corresponding to the trait value of each individual of each species at each site. We considered fire frequency and fire history as fixed effects and species as a random effect. For moisture content and dead biomass, we transformed the data into continuous proportion values and modeled using a beta distribution with a logit link function. For burn rate, we used a Tweedie distribution with a log link function to account for zero values in the data. For maximum temperature and burned biomass, we applied Gaussian distributions with identity links because these variables were approximately normally distributed. Post hoc pairwise comparisons were performed using the R package emmeans (Lenth, 2025) to assess significant differences between fire frequency and fire history interaction using the function emmeans and Tukey method to adjust for multiple comparisons.

To further explore the relationship between biomass conditions (dead biomass and moisture content) and the three components of flammability (burn rate, maximum temperature, and burned biomass), we fitted generalized additive mixed models using the function gamm4 in the R package gamm4 (Wood and Scheipl, 2020). For this analysis, we combined all the observed data, without considering fire frequency or fire history. Each flammability component was modeled as a response variable in relation to smooth terms for dead biomass and moisture content. Species and areas were included as random effects to account for the hierarchical structure of the data and the non‐independence of observations within species or areas. Models were fitted using a Gaussian distribution with an identity link function. The optimal number of basis functions (*k*) for each smooth term was determined through graphical inspection and diagnostic evaluation to balance model complexity and predictive performance.

Model selection was based on the structure of the fixed factors (fire history and fire frequency) using the Akaike information criterion (AIC) to support model construction. We also compared model fit using marginal and conditional *R* ^2^, calculated with the R package performance (Lüdecke et al., 2021). We also calculated Akaike weights (AICw) and ΔAIC to assess the relative support for each model (Appendix S4). Model validation was performed for each model using the function simulateResiduals in the R package DHARMa (Hartig, 2024). Although model selection helped us evaluate the contribution of each factor, we ultimately retained the model with the interaction term (fire history × fire frequency) for interpretation and presentation of results.

All analyses were done in R (R Core Team, 2023), and figures were produced using the R package ggplot2 (Wickham, 2016).

## RESULTS

### Plant community description within study areas

The areas with high fire frequency had the highest species richness, with 93 species where the last fire event was in 2011 and 83 species in the area recently burned in 2019. For these two areas, we measured traits for 12 and 16 species, respectively (Appendix S2). In contrast, the areas with low fire frequency had 56 species where fire was excluded since 2001 and 79 species in the area burned in 2017. We measured traits for 9 and 15 species, respectively (Appendix S2). Within the four study areas, we recorded a 179 unique species, counting shared species once.

Grasses were the dominant growth form, occupying around 85% of the total ground in all areas, while non‐graminoid species (forbs and shrubs) occupied approximately 15%. In all plant communities, *Mesosetum ferrugineum* was the most abundant grass species, occupying about 27% of the ground (Appendix S2).

### Flammability‐related traits according to fire frequency and fire history

**Figure 2:**
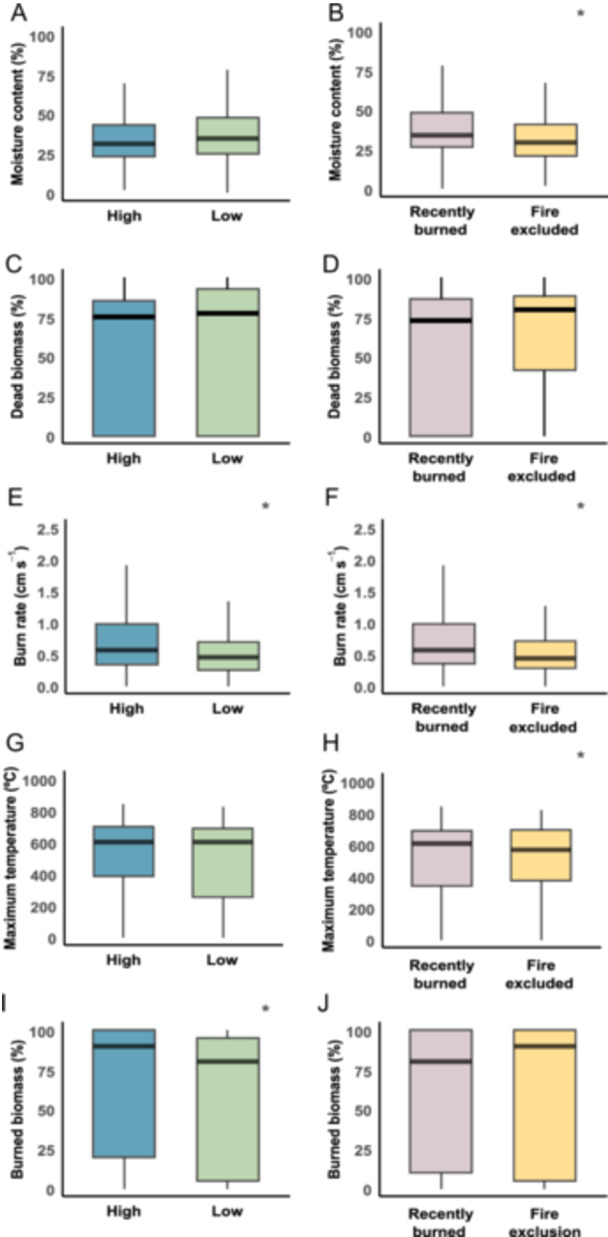
Flammability‐related plant traits in relation to fire frequency (A, C, E, G, I) and fire history (B, D, F, H, J) sampled in areas of open savannas in the Cerrado. Box plot shows the median and first and third quartiles. (*) means statistical differences between high vs. low and recently burned vs. fire‐excluded areas.

Plants had approximately 35% moisture content across both fire frequencies (*z* = 0.58; *P* = 0.56; Figure 2A; Appendix S5A). Regarding fire history, plants in fire‐excluded areas had less moisture than those in recently burned areas (*z* = –4.12; *P* < 0.0001; Figure 2B; Appendix S5B). However, we detected a significant interaction between fire frequency and fire history (*z* = 2.75; *P* = 0.005; conditional $R_{c}^{2}$ = 0.79; $R_{m}^{2}$ = 0.08), indicating that the effect of fire history on plant moisture differed between fire frequencies. Specifically, in fire‐excluded areas, plants under low fire frequency had significantly higher moisture content than those under high fire frequency (*z* = –3.74; *P* = 0.001; Appendix S5C). Plants in both fire frequencies accumulated similar amounts of dead biomass (*z* = 0.04; *P* = 0.96; Figure 2C; Appendix S5A) and fire history (*z* = –0.13; *P* = 0.89; Figure 2D; Appendix S5B), and no interaction was found between the two (*z* = –0.21; *P* = 0.83; Appendix S5C; $R_{c}^{2}$ = 0.99; $R_{m}^{2}$ = 0.001).

As expected, plants had a lower burn rate in low fire frequency areas compared to high fire frequency areas (*z* = –3.45; *P* = 0.0005; Figure 2E; Appendix S5A), burning at 0.5 cm s^–1^. Plants also burned slower in fire‐excluded areas (0.5 ± 0.04 cm s^–1^) compared to plants in recently burned areas (0.75 ± 0.06 cm s^–1^; *z* = –5.58; *P* < 0.0001; Figure 2F). Plants in the two fire frequency groups reached the same maximum temperatures, around 500°C (*z* = –1.59; *P* = 0.11; Figure 2G; Appendix S5A). However, in relation to fire history, plants reached higher maximum temperatures in recently burned areas compared to fire‐excluded areas (*z* = –2.16; *P* = 0.03; Figure 2H; Appendix S5B).

For burned biomass, plants had more than 60% of their biomass consumed by fire in high fire frequency areas, while less than 60% was consumed in low fire frequency (*z* = –2.04; *P* = 0.04; Figure 2I; Appendix S5A). The same amount of biomass was consumed by fire according to fire history (*z* = –1.35; *P* = 0.17; Figure 2J; Appendix S5B). No significant interactions were found for burn rate (*z* = 1.57; *P* = 0.11; Appendix 5C; $R_{c}^{2}$ = 0.62; $R_{m}^{2}$ = 0.1), maximum temperature (*z* = 0.53; *P* = 0.59; Appendix S5C; $R_{c}^{2}$ = 0.7; $R_{m}^{2}$ = 0.01), or burned biomass (*z* = –0.95; *P* = 0.34; Appendix S5C; $R_{c}^{2}$ = 0.81; $R_{m}^{2}$ = 0.02).

### Plant biomass conditions in relation to flammability components

**Figure 3:**
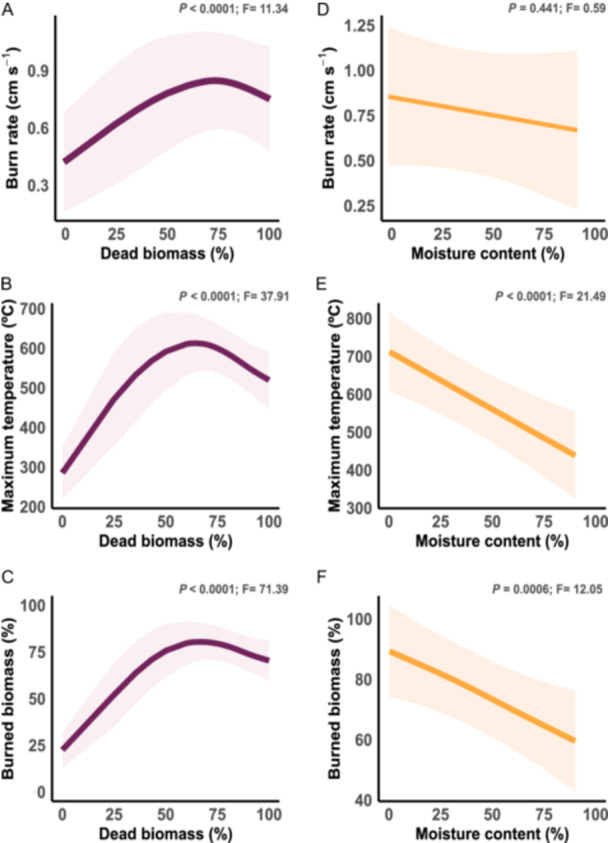
Plant dead biomass and moisture content in relation to burn rate, maximum temperature, and burned biomass. (A–C) Dead biomass vs. burn rate, maximum temperature, and burned biomass, respectively. (D–F) Moisture content vs. burn rate, maximum temperature, and burned biomass, respectively. Solid lines are the predicted values from the GAMM models; smooths are the 95% confidence interval.

We considered plant dead biomass and moisture content in relation to burn rate, maximum temperature, and burned biomass. The amount of dead biomass similarly affected all three flammability components (Figure 3A–C), while moisture content influenced just maximum temperature and burned biomass components (Figure 3E–F).

Plants with less than 50% dead biomass burned slowly (<0.5 cm s^–1^), whereas those with approximately 75% dead biomass had the highest burn rate (>0.8 cm s^–1^). Plants with more than 75% dead biomass had a lower burn rate (Figure 3A). We observed a similar pattern for maximum temperature and burned biomass in relation to burn rate. Plants with less than 50% dead biomass did not reach temperatures above 550°C, while those with approximately 75% dead biomass reached the highest maximum temperatures (600°C). Plants with more than 75% dead biomass reached maximum temperatures below 550°C (Figure 3B). However, plants with less than 50% dead biomass had less than 60% of their biomass consumed by fire, whereas those with more than 75% dead biomass had over 80% of their biomass consumed by fire. Plants with greater dead biomass (>80%) had less than 70% of their biomass consumed by fire (Figure 3C).

The moisture content in plants did not affect burn rate (Figure 3D). However, plants with more moisture had significantly lower maximum temperatures during the burning process (Figure 3E) and less biomass consumed by fire (Figure 3F).

## DISCUSSION

Our study demonstrated that the absence of fire reduced the burn rate of plant communities in the studied open savannas of the Cerrado; the plants burned slower. In addition, our results showed that plants burned differently in relation to fire history, and the amount of biomass consumed differed based on the fire frequency. We found nonlinear relationships between dead biomass and flammability components (burn rate, maximum temperature, and amount of burned biomass), indicating that excessive dead biomass does not necessarily result in greater combustibility and consumability in these areas of open savannas. Furthermore, increasing moisture content consistently decreased maximum temperature and burned biomass, confirming that greater moisture was associated with lower flammability. These findings highlight fire's critical role in maintaining plant biomass conditions that sustain the overall flammability of the herbaceous layer in open savannas of the Cerrado.

Although dead biomass and moisture content are considered important plant traits driving flammability (Simpson et al., 2016; Zanzarini et al., 2022), the fire regime characteristics of our plant communities had minimal influence on these traits. This minimal influence suggests that we may need to consider combining our measured traits with other plant characteristics such as plant architecture and biomass density, which also influence the combustibility and consumability of plants (Simpson et al., 2016; Pausas et al., 2017). In tropical savannas, fire usually promotes tall and upright grasses, facilitating fire consumability (Hempson et al., 2019). Conversely, in the first years of fire exclusion, grasses that accumulate large amounts of dead biomass at their bases start to dominate the ground, suppressing the tall and upright grasses (Rodrigues and Fidelis, 2022). In our studied sites, *Mesosetum ferrugineum* was the most abundant grass species, which certainly influences the system's flammability. Such dense biomass accumulated by those species creates greater moisture content, negatively influencing the components of flammability (Simpson et al., 2016; Pausas et al., 2017; Zanzarini et al., 2022). High biomass density is associated with low rates of fire spread (Prior et al., 2017), due to a limited supply of oxygen (Scarff and Westoby, 2006; Varner et al., 2015). Thus, the reduction in burn rate in low fire frequency and fire‐excluded areas may result from synergistic trait combinations, such as water content, the amount of dead biomass and biomass density, and plant architecture, which directly affect the combustibility of tropical savannas.

Although we found some significant differences in relation to maximum temperature across fire history and for burned biomass across fire frequency, these differences may not be biologically meaningful (e.g., the difference in maximum temperature across fire history was just 4°C, and the difference in burned biomass between fire frequencies was just 5%). Such results reveal that the herbaceous layer is still equally well consumed by fire in these open savannas with different fire regime characteristics. Despite the fact that our low fire frequency areas had just one and two fires in the last 21 years—and one area had its last fire in 2001—the herbaceous layer remains the main vegetation component. However, such plants spend a long time being consumed by fire with hot temperatures (approximately 500°C), which could affect the fire behavior in these fire‐excluded areas (Zupo et al., 2022). Higher fire intensity and severity, due to previous fire suppression, can negatively affect plants, especially shallow‐rooted species, because plant tissues may not tolerate high temperatures for a long time (Bond and Keane, 2017). Thus, these findings confirm that species can burn in fire‐excluded areas, although they typically burn more slowly, which affects fire behavior and plant community dynamics.

When examining the relationship between plant dead biomass and flammability components, we observed that dead biomass increased flammability up to a certain threshold—contrary to our initial hypothesis. When the initial dead biomass surpassed 70%, plants had reduced combustibility and consumability. This pattern aligns with our finding that plants burned more slowly in low fire frequency and fire‐excluded areas. This nonlinear relationship between dead material and flammability components was also detected in *Ulex europaeus*, a European invasive species in New Zealand that dominates natural areas and is highly flammable (Dent et al., 2019). Studies suggest that the most flammable group within a plant community drives the overall flammability (de Magalhães and Schwilk, 2012; Wyse et al., 2018). Notably, grasses accounted for over 75% of the plant community abundance in our study (Appendix S2). However, grasses accumulate more dead biomass compared to forbs and shrubs (Rodrigues and Fidelis, 2022; Zanzarini et al., 2022). Thus, the nonlinear relationship between dead biomass and burn rate, maximum temperature, and burned biomass may be attributed to grass tussocks with accumulated dead biomass in areas undergoing shifts in their fire regimes.

Consistent with our expectations, higher moisture content in plants reduced all flammability components (burn rate, maximum temperature, and burned biomass). Beyond grasses, we also observed a high diversity of less‐flammable plants in the herbaceous layer of the Brazilian open savannas, including forbs and shrubs (Coutinho, 1982; Zanzarini et al., 2022). Those species have traits associated with lower flammability, such as high moisture content and low dead biomass amounts (Zanzarini et al., 2022), which directly influence the measured flammability components, especially maximum temperature and burned biomass. Similarly, in North American tallgrass prairies, grass‐dominated areas burn more completely and at higher temperatures than forb‐dominated areas (Wragg et al., 2018). Thus, the presence of a diverse herbaceous layer in open savannas of the Cerrado, promoted by frequent fires (Rodrigues and Fidelis, 2022), also helps to maintain the common fire regime characteristics of this tropical savanna, which is known to have cool fires (Archibald et al., 2013).

In conclusion, although our study is limited to a single region, with few sites grouped based on fire frequency and history—which do not represent a true gradient of these conditions—our results indicate that, although fire exclusion negatively affects flame propagation through the plants, biomass consumption by fire remains similar to that in regularly and recently burned areas in the open savannas of the Cerrado. In addition, the amount of dead biomass does not have linear relationships with flammability components, indicating that areas with more dead biomass can have their flammability reduced. It is important to state that investigation into flammability patterns should consider a combination of traits that can give a better understanding about how fire‐prone ecosystems burn. Nevertheless, it is also important to investigate how flammability‐related traits may vary in plant communities under different fire regimes and incorporate this information into fire models (Simpson et al., 2022), contributing to improving fire management policies. Thus, we need to consider that changes in local fire regimes will affect how tropical savannas burn.

## AUTHOR CONTRIBUTIONS

V.Z. and A.F. designed the study and collected the data. D.R. contributed to the written and results/analyses discussions. All authors wrote and revised the text.

## Supporting information


**Appendix S1.** Fire history of the four selected areas, which varied in fire frequency (high, low) and year since the last fire (2019, 2017, 2011, 2001).

**Appendix S2.** Abundance of the selected species in the four studied areas of open savanna in the Cerrado.

**Appendix S3.** (A) Eequipment constructed to measure flammability components.

**Appendix S4.** Model comparison results for each response variable.

**Appendix S5.** Means for flammability‐related traits in relation to fire frequency and fire history.

## Data Availability

The data that support the study are available at Zenodo: https://doi.org/10.5281/zenodo.17086311.
