# Supplementary material for: Changes in plant flammability‐related traits to fire regime characteristics and biomass conditions in the Cerrado
Source: Am J Bot. 2025 Oct 14;112(10):e70110. doi: 10.1002/ajb2.70110 (PMC12572697; doi:10.1002/ajb2.70110)
Supplement: Supplementary file 1 — Appendix S1. Fire history of the four selected areas, which varied in fire frequency (high, low) and year since the last fire (2019, 2017, 2011, 2001). [file AJB2-112-e70110-s002.docx]

**Appendix S1.** Fire history of the four selected areas, which vary in fire frequency (high, low) and year since the last fire (2019, 2017, 2011, and 2001).

| **Area** | **Coordinates** | **Year of fire occurrence**  **(2000–2021)** | **Time since the last fire (yr)** | **Number of fires** |
| --- | --- | --- | --- | --- |
| **High, last fire in 2019** | 13°40'53'S 47°48'38''W | 2001, 2002, 2003, 2004, 2005, 2007, 2011, 2013, 2015, 2017, 2019 | 2 | 11 |
| **High, last fire in 2011** | 13°40'50''S 47°48'46'W | 2001, 2002, 2003, 2004, 2005, 2007, 2011 | 13 | 7 |
| **Low, last fire in 2017** | 13°39'05''S 47°49'54''W | 2001, 2017 | 4 | 2 |
| **Low, last fire in 2001** | 13°39'26''S 47°49'28''W | 2001 | 20 | 1 |
