# Supplementary material for: Changes in plant flammability‐related traits to fire regime characteristics and biomass conditions in the Cerrado
Source: Am J Bot. 2025 Oct 14;112(10):e70110. doi: 10.1002/ajb2.70110 (PMC12572697; doi:10.1002/ajb2.70110)
Supplement: Supplementary file 2 — Appendix S2. Abundance of the selected species in the four studied areas of open savanna in the Cerrado. [file AJB2-112-e70110-s003.docx]

**Appendix S2.** Abundance (% cover) of the selected species in the four areas of open savannas in the Cerrado. Areas were classified by fire frequency (high, low) and fire history (2019, 2017, 2011, 2001).

| **Growth form** | **Family** | **Species** | **High, last fire 2019** | **High, last fire 2011** | **Low, last fire 2017** | **Low, last fire 2001** |
| --- | --- | --- | --- | --- | --- | --- |
| Grass | Poaceae | *Anthaenantia lanata* (Kunth) Benth. | 2.73 | 0 | 2.06 | 5.24 |
|  | Poaceae | *Andropogon lateralis* Ness | 2.54 | 0 | 1.31 | 0 |
|  | Poaceae | *Aristida setifolia* Kunth | 2.29 | 5.08 | 0 | 0 |
|  | Poaceae | *Arthropogon villosus* Nees | 0 | 14.13 | 0 | 2.32 |
|  | Poaceae | *Axonopus aureus* P. Beauv. | 0 | 0 | 0 | 2.98 |
|  | Poaceae | *Elionurus muticus* (Spreng.) Kuntze | 14.18 | 2.51 | 3.36 | 0 |
|  | Poaceae | *Mesosetum ferrugineum* (Trin.) Chase | 28.86 | 30.41 | 35.13 | 16.14 |
|  | Poaceae | *Mesosetum loliiforme* (Hochst.) Chase | 3.20 | 3.09 | 8.48 | 39.37 |
|  | Poaceae | *Oncorachis ramosa* (Zuloaga & Soderstr.) Morrone & Zuloaga | 4.89 | 19.39 | 7.29 | 2.38 |
|  | Poaceae | *Paspalum gardnerianum* Nees | 0 | 0 | 1.49 | 0 |
|  | Poaceae | *Paspalum pectinatum* Nees ex Trin*.* | 5.02 | 5.36 | 0 | 0 |
|  | Poaceae | *Paspalum thrasyoides* (Trin.) S. Denham | 0 | 0 | 0 | 20.19 |
|  | Poaceae | *Trachypogon spicatus* (L.f.) Kuntze | 5.55 | 0.00 | 27.65 | 9.59 |
|  | Poaceae | *Trichanthecium cyanescens* (Nees ex Trin.) Zuloaga & Morrone | 0 | 2.45 | 0 | 0 |
|  | Poaceae | *Trichanthecium* sp. | 0 | 0 | 3.25 | 0 |
| **Total grass cover** | |  | **69.26** | **82.42** | **90.02** | **98.21** |
| Forb | Euphorbiaceae | *Croton gracilescens* Müll. Arg. | 5.99 | 5.20 | 1.87 | 0 |
|  | Asteraceae | *Ayapana amygdalina* (Lam.) R.M. King & H. Rob. | 0 | 0 | 1.49 | 0 |
|  | Gentianaceae | *Calolisianthus speciosus* (Cham. & Schltdl.) Gilg | 0 | 0 | 1.23 | 0 |
|  | Fabaceace | *Chamaecrista ochrosperma* (H.S. Irwin & Barneby) | 2.98 | 0 | 0 | 0 |
|  | Asteraceae | *Ichthyothere hirsuta* Gardner | 1.69 | 0 | 0 | 0 |
|  | Acanthaceae | *Ruellia trachyphylla* Lindau | 4.36 | 1.69 | 0 | 0 |
|  | Vochysiaceae | *Vochysia pumila* Pohl | 0 | 0 | 2.47 | 0 |
| **Total forb cover** | |  | **15.03** | **6.90** | **7.06** | **0** |
| Shrub | Fabaceace | *Bauhinia dumosa* Benth. | 5.11 | 4.33 | 1.35 | 0 |
|  | Moraceae | *Brosimum gaudichaudii* Trécul | 0 | 0 | 0 | 1.79 |
|  | Fabaceace | *Calliandra dysantha* Benth*.* | 1.98 | 0 | 1.57 | 0 |
|  | Fabaceace | *Mimosa pteridifolia* Benth. | 8.63 | 6.35 | 0 | 0 |
| **Total shrub cover** | |  | **15.72** | **10.68** | **2.91** | **1.79** |
