# Supplementary material for: Changes in plant flammability‐related traits to fire regime characteristics and biomass conditions in the Cerrado
Source: Am J Bot. 2025 Oct 14;112(10):e70110. doi: 10.1002/ajb2.70110 (PMC12572697; doi:10.1002/ajb2.70110)

**Appendix S3.** Equipment for measuring flammability components, constructed as described by Jaureguiberry et al. (2011). (A) An 85 × 60 cm barrel was cut longitudinally and placed on a metal support with four legs. The barrel was open at the front and closed at the back for wind protection. Samples were arranged horizontally on a grill grate and heated by a burner beneath the grill. (B) Sample burning during the flammability measurements.


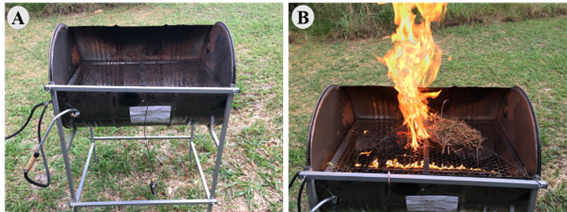

Supplement: Supplementary file 3 — Appendix S3. (A) Eequipment constructed to measure flammability components. [file AJB2-112-e70110-s004.docx]
