# Supplementary material for: Changes in plant flammability‐related traits to fire regime characteristics and biomass conditions in the Cerrado
Source: Am J Bot. 2025 Oct 14;112(10):e70110. doi: 10.1002/ajb2.70110 (PMC12572697; doi:10.1002/ajb2.70110)
Supplement: Supplementary file 4 — Appendix S4. Model comparison results for each response variable. [file AJB2-112-e70110-s001.docx]

**Appendix S4.** Model comparison results for each response variable, including the corrected Akaike information criterion (AICc), ΔAICc, Akaike weights (wAICc), and marginal and conditional *R*² values. ΔAICc refers to the difference in AICc units between a given model and the most parsimonious model in the candidate set. wAICc represents the Akaike weight, indicating the relative probability that a given model is the best among those compared. Marginal $R_{m}^{2}$reflects the variance explained by fixed effects only; conditional $R_{c}^{2}$includes both fixed and random effects.

| **Model** | | **df** | **AICc** | **ΔAICc** | **wAICc** | $\boldsymbol{R}_{\mathbf{c}}^{\boldsymbol{2}}$ | $\boldsymbol{R}_{\mathbf{m}}^{\boldsymbol{2}}$ |
| --- | --- | --- | --- | --- | --- | --- | --- |
| null | moisture cont. ~ 1 + (1 \| species) | 3 | -357.21 | 18.71 | 0.00 | 0.75 | 0.00 |
| 1 | moisture cont. ~ fire history * frequency + (1 \| species) | 6 | -375.91 | 0.00 | 0.92 | 0.79 | 0.08 |
| 2 | moisture cont. ~ fire history + frequency + (1 \| species) | 5 | -370.61 | 5.31 | 0.07 | 0.76 | 0.06 |
| 3 | moisture cont. ~ fire history + (1 \| species) | 4 | -366.67 | 9.25 | 0.01 | 0.76 | 0.04 |
| 4 | moisture cont. ~ frequency + (1 \| species) | 4 | -363.49 | 12.42 | 0.00 | 0.76 | 0.04 |
|  | |  | |  |  |  | |
| null | dead biomass ~ 1 + (1 \| species) | 3 | -1050.63 | 0.00 | 0.52 | 0.99 | 0.001 |
| 1 | dead biomass ~ fire history * frequency + (1 \| species) | 7 | -1044.54 | 6.09 | 0.02 | 0.99 | 0.001 |
| 2 | dead biomass ~ fire history + frequency + (1 \| species) | 6 | -1046.59 | 4.04 | 0.07 | 0.99 | 0.001 |
| 3 | dead biomass ~ fire history + (1 \| species) | 5 | -1048.66 | 1.97 | 0.20 | 0.99 | 0.001 |
| 4 | dead biomass ~ frequency + (1 \| species) | 5 | -1048.57 | 2.06 | 0.19 | 0.99 | 0.001 |
|  | |  | |  |  |  | |
| null | burn rate ~ 1 + (1 \| species) | 5 | 385.15 | 36.56 | 0.00 | 0.56 | 0.00 |
| 1 | burn rate ~ fire history * frequency + (1 \| species) | 8 | 348.59 | 0.00 | 0.54 | 0.62 | 0.10 |
| 2 | burn rate ~ fire history + frequency + (1 \| species) | 7 | 348.93 | 0.34 | 0.45 | 0.64 | 0.11 |
| 3 | burn rate ~ fire history + (1 \| species) | 6 | 356.41 | 7.82 | 0.01 | 0.65 | 0.07 |
| 4 | burn rate ~ frequency + (1 \| species) | 6 | 379.49 | 30.90 | 0.00 | 0.55 | 0.03 |
|  | |  | |  |  |  | |
| null | max. temp. ~ 1 + (1 \| species) | 3 | 3429.22 | 2.95 | 0.09 | 0.70 | 0.00 |
| 1 | max. temp. ~ fire history * frequency + (1 \| species) | 6 | 3428.08 | 1.81 | 0.15 | 0.70 | 0.01 |
| 2 | max. temp. ~ fire history + frequency + (1 \| species) | 5 | 3426.27 | 0.00 | 0.37 | 0.71 | 0.01 |
| 3 | max. temp. ~ fire history + (1 \| species) | 4 | 3426.61 | 0.34 | 0.32 | 0.71 | 0.01 |
| 4 | max. temp. ~ frequency + (1 \| species) | 4 | 3429.50 | 3.23 | 0.07 | 0.69 | 0.00 |
|  | |  | |  |  |  | |
| null | burned biomass ~ 1 + (1 \| species) | 3 | 3429.22 | 1055.78 | 0.00 | 0.70 | 0.00 |
| 1 | burned biomass ~ fire history * frequency + (1 \| species) | 6 | 2374.63 | 1.19 | 0.32 | 0.81 | 0.02 |
| 2 | burned biomass ~ fire history + frequency + (1 \| species) | 5 | 2373.44 | 0.00 | 0.58 | 0.80 | 0.02 |
| 3 | burned biomass ~ fire history + (1 \| species) | 4 | 2380.84 | 7.40 | 0.01 | 0.79 | 0.00 |
| 4 | burned biomass ~ frequency + (1 \| species) | 4 | 2377.23 | 3.79 | 0.09 | 0.79 | 0.01 |
