# Supplementary material for: Changes in plant flammability‐related traits to fire regime characteristics and biomass conditions in the Cerrado
Source: Am J Bot. 2025 Oct 14;112(10):e70110. doi: 10.1002/ajb2.70110 (PMC12572697; doi:10.1002/ajb2.70110)
Supplement: Supplementary file 5 — Appendix S5. Means for flammability‐related traits in relation to fire frequency and fire history. [file AJB2-112-e70110-s005.docx]

**Appendix S5.** Means ± SE for plant flammability-related traits in relation to fire frequency and fire history.

**(A)** Means ± SE in relation to fire frequency.

| **Fire frequency** | **Moisture conten**t (%) | **Dead**  **biomass (%)** | **Burn rate**  **(cm s^–1^) *** | **Maximum temperature (ºC)** | **Burned biomass (%) *** |
| --- | --- | --- | --- | --- | --- |
| **High** | 33.50 ± 1.26 | 52.30 ± 3.39 | 0.77 ± 0.06 | 516.58 ± 20.35 | 64.75 ± 3.46 |
| **Low** | 37.41 ± 1.50 | 60.43 ± 3.61 | 0.50 ± 0.04 | 473.53 ± 24.93 | 58.55 ± 3.75 |

* Statistical significance between fire frequencies.

**(B)** Means ± SE in relation to fire history.

| **Fire history** | **Moisture content (%) *** | **Dead**  **biomass (%)** | **Burn rate**  **(cm s^–1^) *** | **Maximum temperature (ºC) *** | **Burned biomass (%)** |
| --- | --- | --- | --- | --- | --- |
| **Recently burned** | 38.25 ± 1.35 | 51.54 ± 3.37 | 0.75 ± 0.06 | 498.78 ± 20.59 | 61.10 ± 3.30 |
| **Fire exclusion** | 31.18 ± 1.29 | 62.55 ± 3.55 | 0.50 ± 0.04 | 493.16 ± 25.28 | 62.92 ± 4.01 |

* Statistical significance between fire frequencies.

**(C)** Means ± SE for traits in each of the four areas of open savannas in the Cerrado. Areas are identified based on fire frequency (high/low) and fire history (2019, 2017, 2011, 2001).

| **Area** | **Moisture content (%)** | **Dead biomass (%)** | **Burn rate (cm s^–1^)** | **Maximum temperature (°C)** | **Burned biomass (%)** |
| --- | --- | --- | --- | --- | --- |
| **High, last fire 2019** | 36.38 ± 1.67 | 50.38 ± 4.55 | 0.96 ± 0.09 | 547.94 ± 23.91 | 68.20 ± 4.29 |
| **High, last fire 2011** | 29.71 ± 1.82 | 54.82 ± 5.12 | 0.53 ± 0.05 | 475.27 ± 34.63 | 60.20 ± 5.66 |
| **Low, last fire 2017** | 40.25 ± 2.13 | 52.78 ± 5.03 | 0.53 ± 0.05 | 446.29 ± 33.16 | 53.51 ± 4.92 |
| **Low, last fire 2001** | 33.02 ± 1.80 | 72.22 ± 4.48 | 0.46 ± 0.05 | 515.52 ± 37.07 | 66.31 ± 5.65 |
